# sLOX-1: A Molecule for Evaluating the Prognosis of Recurrent Ischemic Stroke

**DOI:** 10.1155/2021/6718184

**Published:** 2021-08-28

**Authors:** Yangmin Zheng, Yuyou Huang, Lingzhi Li, Pingping Wang, Rongliang Wang, Zhen Tao, Junfen Fan, Ziping Han, Fangfang Li, Haiping Zhao, Fangfang Zhao, Feng Yan, Yumei Liu, Yumin Luo

**Affiliations:** ^1^Institute of Cerebrovascular Disease Research and Department of Neurology, Xuanwu Hospital of Capital Medical University, Beijing 100000, China; ^2^Beijing Geriatric Medical Research Center and Beijing Key Laboratory of Translational Medicine for Cerebrovascular Diseases, Beijing 100000, China; ^3^Vascular Ultrasonography Department, Xuanwu Hospital, Capital Medical University, Beijing 100000, China

## Abstract

Several clinical parameters and biomarkers have been proposed as prognostic markers for stroke. However, it has not been clarified whether the risk factors affecting the prognosis of patients with recurrent and first-ever stroke are similar. In this study, we aimed to explore the relationship between soluble lectin-like oxidized low-density lipoprotein receptor 1 (sLOX-1) levels and the prediction of the functional outcome in patients with recurrent and first-ever stroke. A total of 266 patients with recurrent and first-ever stroke, who underwent follow-up for 3 months, were included in this study. Plasma samples were collected within 24 h after onset. The results showed that biomarkers for the prognosis of patients with recurrent stroke were different from that of those with first-ever stroke. sLOX-1 levels were correlated with modified Rankin Scale scores of patients with recurrent stroke alone (*r* = 0.3232, *p* = 0.001). sLOX-1 levels were also associated with an increased risk of unfavorable outcomes in patients with recurrent stroke with an adjusted odds ratio of 1.489 (95% confidence interval, 1.204–1.842, *p* < 0.0001). Combining the risk factors showed greater accuracy for prognosis, yielding a sensitivity of 93.2% and a specificity of 75%, with an area under the curve of 0.916, evaluated by the receiver operating characteristic curve. These findings suggest that the diagnosis and prognosis are different between patients with recurrent stroke and those with first-ever stroke, and sLOX-1 level is an independent prognostic marker in patients with recurrent stroke.

## 1. Introduction

Stroke is a type of cerebrovascular disorder with high morbidity, disability, mortality, and recurrence rate. Its consequences are severe, especially after recurrence [1]. There are differences in the treatment and rehabilitation between patients with first-ever stroke and those with recurrent stroke [2]. Patients with previous stroke have a significantly increased risk of recurrence, and their severe disability and mortality rates are significantly higher [3]. However, it has not been clarified whether the risk factors of recurrent and first-ever stroke are completely similar, and there are few studies on the severity and short-term prognosis of recurrent ischemic stroke. Therefore, it is important to explore the risk factors affecting the prognosis of patients with recurrent ischemic stroke and to assess the condition and short-term prognosis of patients with recurrent stroke to improve their quality of life [4]. In addition, after screening patients with a history of stroke during clinical diagnosis and treatment, the direction of future research is to determine whether individualized treatment can be provided.

Lectin-like oxidized low-density lipoprotein receptor 1 (LOX-1), a type II integrin membrane glycoprotein receptor, is an acute-phase reactant, usually with low basal expression levels in the cells. Its expression increases rapidly due to various prooxidation and proinflammatory cytokines [5, 6]. Therefore, it plays an important role in oxidative stress and cell damage induced by inflammatory factors. Soluble LOX-1 (sLOX-1) is a proteolytic form of LOX-1 that is released from the plasma membrane into the extracellular circulation after cell damage [7]. Clinical studies have shown that increased sLOX-1 levels might be positively correlated with intracranial artery stenosis in patients with stroke [8–12]. Furthermore, plasma sLOX-1 levels are also associated with the risk of carotid plaque inflammation and occurrence of ischemic stroke [13]. Therefore, plasma sLOX-1 levels can be used to evaluate the severity of stroke and intracranial arterial stenosis. Studies have shown that inflammatory factors enhance sLOX-1 cleavage in tumor necrosis factor- (TNF-) activated cultured endothelial cells and LOX-1 transgenic mice in vivo [14]. Meanwhile, clinical studies have shown that increased sLOX-1 levels are positively correlated with various inflammatory markers such as TNF-*α* [15]; it is possible to use sLOX-1 levels to predict the functional prognosis of stroke. Plasma sLOX-1 levels may be a novel potential biomarker for predicting the risk for multiple subtypes of stroke [9–11, 16–18]. Serum sLOX-1 level was higher in patients with large artery atherosclerotic (LAA) stroke and is used as a biomarker in patients with LAA ischemic stroke [16]. Serum sLOX-1, which is positively associated with hemorrhagic severity, may have the potential to be a biomarker for delayed cerebral ischemia after aneurysmal subarachnoid hemorrhage [19]. sLOX-1 levels may also be a potential biomarker for predicting the risk for acute ischemic stroke (AIS) in patients with internal carotid artery stenosis [9]. In addition, sLOX-1 can be used to predict the long-term prognosis of AIS [11]. However, the role of sLOX-1 in AIS remains unclear, especially between recurrent ischemic stroke and first-ever ischemic stroke. Therefore, this study enrolled a total of 266 patients with AIS, including 101 with recurrent ischemic stroke and 165 with first-ever ischemic stroke, to evaluate the relationship between sLOX-1 levels and the prediction of functional outcomes in patients with AIS.

## 2. Materials and Methods

### 2.1. Study Participants

**Figure 1:**
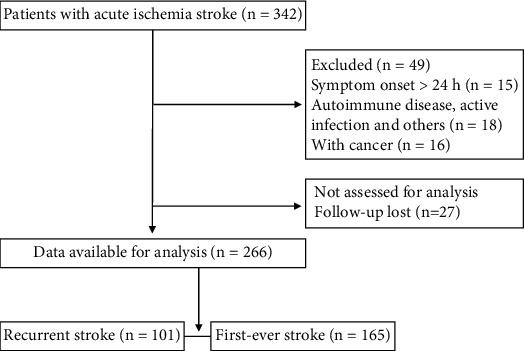
Study flowchart for participant selection.

This study was reviewed and approved by the Ethics Committee of the Institutional Review Board of Capital Medical University, Beijing, China and was in accordance with the Declaration of Helsinki. All patients signed an informed consent form. Two hundred and sixty-six patients with AIS (101 with recurrent ischemic stroke and 165 first-ever ischemic stroke) undergoing follow-up for 3 months at the Cerebrovascular Diseases Research Institute of Xuanwu Hospital of Capital Medical University were included in this study. Basic data were collected within 24 h after the onset of AIS. The study flowchart for participant selection is shown in Figure 1.

The inclusion criteria were as follows: (1) admission within 24 h of symptom onset, (2) AIS confirmed by brain magnetic resonance imaging (MRI) or computed tomography (CT), and (3) recurrent ischemic stroke with a clear history of ischemic stroke and more than 1 month away from onset. In addition, there should be clinical symptoms and signs of new ischemic stroke, and imaging should show new lesions. The exclusion criteria were the following: (1) autoimmune disease, (2) cerebral hemorrhage, (3) transient ischemic attack, and (4) progressive stroke or progressive deterioration of stroke.

### 2.2. Clinical Data

Clinical baseline data were collected after the onset of the disease (including the patient's general admission data, routine blood biochemistry, other hematological examinations, MRI/CT infarct volume, National Institutes of Health Stroke Scale (NIHSS) score, and modified Rankin Scale (mRS)). After admission, the NIHSS score was used to evaluate the degree of neurological impairment. The mRS score at 90 days follow-up was recorded by an experienced neurologist by telephone. An mRS score of 0–2 at follow-up was defined as a favorable outcome and 3–6 as an unfavorable outcome. The boundary of the patients' lesions was measured by using RadiAnt DICOM Viewer on the first brain CT or brain MRI diffusion-weighted imaging sequence after admission. Finally, the lesion area of each layer was multiplied by a thickness of 0.5 cm to obtain the infarct volume layer by layer.

### 2.3. Measurement of Soluble LOX-1

Before treatment, the blood samples of patients with AIS were collected in K3 EDTA tubes. The plasma was separated and frozen at -80°C. The concentrations of sLOX-1 were determined using commercially available enzyme-linked immunosorbent assay kits according to the manufacturer's instructions (Solarbio Life Science, Beijing, China).

### 2.4. Statistical Analyses

All analyses were conducted using SPSS software (version 22.0; IBM Corp., Armonk, NY, USA) and Prism7 software (GraphPad Software Inc., USA). The Kolmogorov–Smirnov test was used to determine whether the data were normally distributed. Continuous variables that were normally distributed were expressed as the mean value ± standard deviation and analyzed using the independent *t*-test, while continuous variables that were not normally distributed were expressed as medians with interquartile ranges (25^th^–75^th^ percentiles) and analyzed using the Mann–Whitney *U* test. Categorical variables were expressed as counts and proportions and were compared using the chi-square test. Variables with *p* values < 0.1, from univariate analyses and variables that were previously reported, were incorporated in the multivariate analysis. Based on the receiver operating characteristic (ROC) curve analysis, the corresponding sensitivity, specificity, and area under the curve (AUC) were calculated to evaluate the accuracy of serum sLOX-1 in predicting the prognosis of AIS. Furthermore, Prism7 software was used to draw a forest plot to show the results of the multivariate regression analysis.

## 3. Results and Discussion

### 3.1. Elevated sLOX-1 Levels Were Correlated with an Increased Risk of Adverse Outcomes in Patients with AIS with Recurrent Ischemic Stroke but Not with First-Ever Stroke

**Figure 2:**
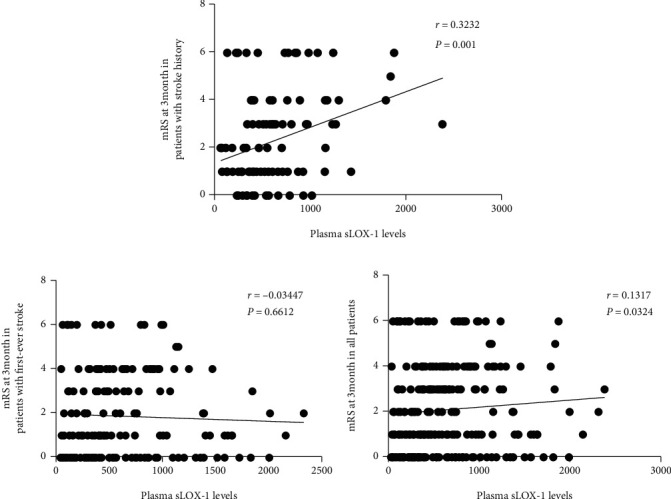
Correlations between sLOX-1 levels and 3-month mRS score. Correlation of 3-month mRS score with the plasma sLOX-1 level in patients with AIS with recurrent ischemic stroke (a), with first-ever stroke (b), and in all patients with AIS (c). *N* = 101 for patients with recurrent ischemic stroke, *N* = 165 for patients with first-ever ischemic stroke, and *N* = 266 for all patients with AIS. AIS: acute ischemic stroke; sLOX-1: soluble lectin-like oxidized low-density lipoprotein receptor-1; mRS: modified Rankin Scale.

Plasma sLOX-1 levels were positively correlated with the mRS score at 3 months in patients with recurrent ischemic stroke, as shown in Figure 2 (*r* = 0.3232, *p* = 0.001). However, no linear correlation was found between sLOX-1 levels and mRS score at 3 months in all patients with AIS or with first-ever stroke.

**Figure 3:**
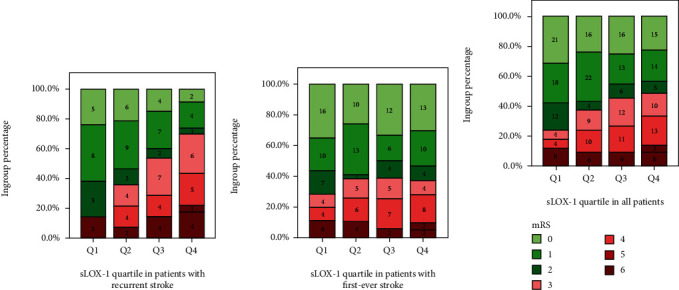
Elevated sLOX-1 levels were correlated with an increased risk of adverse outcomes in patients with recurrent stroke but not in those with first-ever stroke. The proportion of patients with favorable (mRS score = 0–2) and unfavorable outcomes (mRS score = 3–6) in patients with recurrent ischemic stroke (a), with first-ever stroke (b), and in all patients with AIS (c). Grouped according to the quartile of sLOX-1 level: Q1 represents ≤316.72 pg/mL, Q2 represents 316.72–510.52 pg/mL, Q3 represents 510.52–877.48 pg/mL, and Q4 represents ≥877.48 pg/mL. Numbers indicate the number of cases per subgroup.

To further observe this correlation, we grouped sLOX-1 by quartiles.  Figure 3 shows that after being grouped in quartiles of sLOX-1 level the proportion of patients in each quartile group with an unfavorable outcome in patients with AIS with recurrent ischemic stroke showed a gradient increase with increasing sLOX-1 levels. Meanwhile, no such change was observed in patients with first-ever ischemic stroke.

### 3.2. Baseline Characteristics of Patients Grouped by Recurrent Ischemic Stroke and First-Ever Ischemic Stroke

A total of 266 patients with AIS were enrolled in this study. These patients were grouped into having either recurrent ischemic stroke or first-ever ischemic stroke. Their baseline characteristics are shown in Table 1.

**Figure 4:**
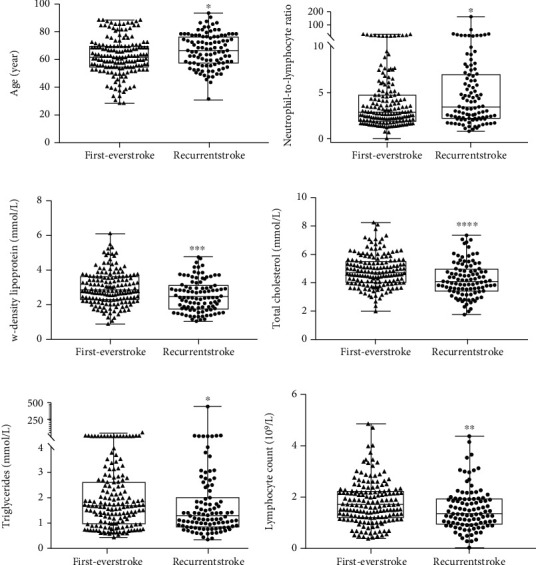
Significant differences were found in baseline data between patients with recurrent stroke and those with first-ever stroke. Patients with first-ever stroke were younger (a), and the neutrophil-to-lymphocyte ratio (b) was higher in patients with recurrent stroke; patients with first-ever stroke had higher low-density lipoprotein content (c), total cholesterol levels (d), total triglyceride levels (e), and lymphocyte count (f) than patients recurrent stroke. ^∗^*p* < 0.05, ^∗∗^*p* < 0.005, and ^∗∗∗^*p* < 0.001.

We found that patients with recurrent ischemic stroke were older than those with first-ever ischemic stroke, as shown in Figure 4(a), although there was no difference in sex composition. We also found that the neutrophil-to-lymphocyte ratio (NLR) of patients with recurrent ischemic stroke was higher than those with first-ever ischemic stroke. Furthermore, we found that lymphocyte count, total cholesterol and triglyceride levels, and low-density lipoprotein content were significantly different, and these indicators were significantly higher in patients with first-ever ischemic stroke than in those with recurrent ischemic stroke, as shown in Figures 4(c)–4(f).

We speculated that the patients with recurrent ischemic stroke received sufficient treatment and rehabilitation after the first-ever stroke, which allowed some stroke risk factors to be controlled. This may also explain why sLOX-1 levels were correlated with the mRS score at 3 months in patients with recurrent stroke but not in those with first-ever stroke. However, the stroke risk factors were not effectively controlled in patients with first-ever stroke before the occurrence of stroke, and the risk factors of stroke are relatively complex; thus, no correlation was found between sLOX-1 levels and mRS score in patients with first-ever stroke.

### 3.3. sLOX-1 Levels Represented an Independent Predictor for Unfavorable Outcomes in Patients with AIS with Recurrent Ischemic Stroke but Not with First-Ever Stroke

We further examined the predictive value of sLOX-1 levels for unfavorable outcomes after recurrent or first-ever stroke. Univariate analyses showed that high sLOX-1 levels were associated with an increased risk of unfavorable outcomes with recurrent ischemic stroke, as shown in Table 2 (*p* < 0.0001), but not with first-ever ischemic stroke.

**Figure 5:**
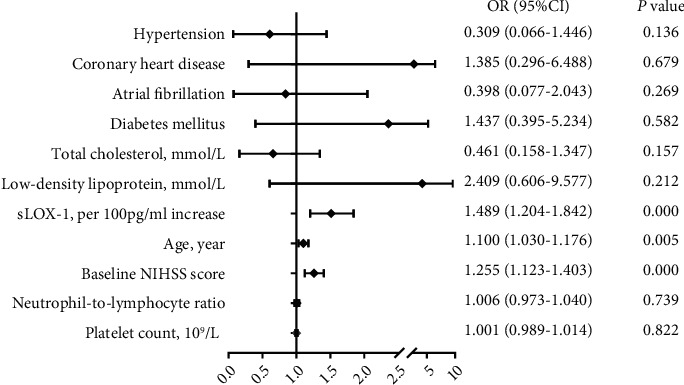
Forest plot shows the risk of the primary outcome after AIS. After adjusting for age, admission NIHSS score, neutrophil-to-lymphocyte ratio, diabetes mellitus, atrial fibrillation, coronary heart disease, hypertension, platelet count, low-density lipoprotein, and total cholesterol, binominal multivariate logistic analysis showed that sLOX-1 level was an independent predictor for unfavorable outcome in patients with recurrent ischemic stroke.

However, there was no significant difference in sLOX-1 levels between patients with recurrent ischemic stroke and those with first-ever stroke (526.36 pg/mL vs. 510.51 pg/mL, *p* = 0.824). In addition, in the univariate analysis, lymphocyte and neutrophil counts and atrial fibrillation were associated with unfavorable outcomes in patients with first-ever stroke, but not in those with recurrent stroke. This suggests that the prognostic biomarkers in patients with recurrent ischemic stroke may be different from those in patients with first-ever stroke. After adjusting for age, admission NIHSS score, NLR, and other variables in the binominal multivariate logistic analysis, sLOX-1 levels remained an independent predictor of unfavorable outcomes in patients with recurrent ischemic stroke with an adjusted odds ratio of 1.489, as shown in Figure 5 (95% confidence interval, 1.204–1.842, *p* < 0.0001).

**Figure 6:**
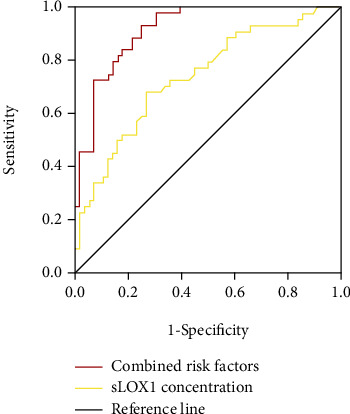
ROC curve analysis on predictive values of age, baseline NIHSS score, sLOX-1 concentration, time from onset, neutrophil count, and combined risk factors. The optimal cutoff value was 575.39 pg/mL, with a sensitivity of 93.2% and a specificity of 75% (AUC: 0.916, 95% confidence interval: 0.863–0.968, *p* < 0.0001).

Based on the ROC curve analysis, the optimal cutoff value of serum sLOX-1 level as an indicator for unfavorable outcome was projected to be 575.39 pg/mL, yielding a sensitivity of 68.2% and a specificity of 73.2%, with an AUC of 0.739. The ROC curves of sLOX-1 levels for the prediction of unfavorable outcomes in patients with recurrent ischemic stroke outcomes are shown in Figure 6 (yellow curve).

We further used the ROC curve to evaluate the diagnostic value of sLOX-1 levels for the prognosis of the subjects in combination with variables of the binominal multivariate logistic analysis. Compared with any of the variables of the binominal multivariate logistic analysis or sLOX-1 levels alone, the combination of sLOX-1 and the variables showed greater accuracy, yielding a sensitivity of 93.2%, positive predictive value of 93.3%, negative predictive value of 74.5%, specificity of 75%, diagnostic accuracy of 83%, and an AUC of 0.916 (Figure 6, red curve).

## 4. Discussion

This is the first study to show that biomarkers for prognosis are different in recurrent and first-ever strokes. Previous studies have focused on the risk factors for the occurrence and prognosis of first-ever stroke, although these studies have ignored the fact that the prognostic risk factors for recurrent ischemic stroke may be different from those for first-ever stroke. We found that elevated sLOX-1 levels were correlated with an increased risk of adverse outcomes in patients with AIS with recurrent ischemic stroke, but not in those with first-ever stroke. With this, sLOX-1 levels might be used as an indicator for risk stratification and prognosis assessment of patients with recurrent ischemic stroke.

Recurrent ischemic stroke occurs in relation to the first-ever stroke. Brain tissue ischemia and hypoxia cause irreversible damage to some nerve functions. Under the combined action of multiple risk factors, recurrent stroke causes the brain tissue to suffer from hypoperfusion and oxidative stress again, further aggravating the nerve injury [20]. However, it has not been clarified whether the risk factors for recurrent and first-ever stroke are completely similar, and there are few studies on the severity and short-term prognosis of recurrent ischemic stroke. LOX-1 is involved in endothelial cell dysfunction, monocyte proliferation, adhesion and migration, platelet activation, and other inflammatory responses [21]. The sLOX-1 levels can effectively reflect the expression of LOX-1 in the organism [22]. Therefore, it is of great importance to study the role of sLOX-1 in cardiovascular and cerebrovascular diseases. Here, we found that sLOX-1 concentrations were positively correlated with the mRS score at 3 months in patients with recurrent ischemic stroke, and the poor prognosis proportion of the top quartile of the sLOX-1 index was higher than that of the low-level groups. Several known stroke risk factors, such as total cholesterol levels, triglycerides, and low-density lipoprotein content, are lower in patients with recurrent stroke than in patients with first-ever stroke. Therefore, we speculated that many patients had other underlying diseases, for which they did not receive treatment, before AIS occurred, and subsequent changes in the blood sample indexes affected the assessment for the real cause of stroke. Patients with a history of stroke were found to have other diseases, such as hyperlipidemia, at the first stroke attack and were then managed with the corresponding treatment. Thus, molecular changes, due to the treatment and control of other basic diseases, in the plasma samples of patients with a history of stroke may be more able to reflect the direct factors of stroke recurrence.

Current studies on sLOX-1 and other molecules as potential prognostic markers do not consider the patient's history of stroke (or not mentioned) [9–11, 17] or may have already excluded patients with a history of stroke upon enrollment [12, 16, 19]. Therefore, information on the incidence of stroke-related molecules or markers may be missing. Since recurrent ischemic stroke occurs relative to the initial ischemic stroke, it is speculated that both have similar risk factors. To identify the risk factors affecting the prognosis of recurrent ischemic stroke, univariate and multivariate logic analyses were conducted on the established risk factors of initial ischemic stroke in patients with recurrent ischemic stroke and found that age, NIHSS scores, and sLOX-1 levels could affect the prognosis. However, in our study, the sLOX-1 level was not a marker of prognosis in patients with first-ever stroke, which is different from previous findings. We speculated that this might be due to the different receiving times of the plasma samples. The plasma samples of the patients in our study were collected within 24 h of the onset of stroke, while those in other studies were different; some were collected within 72 h [11] and others within 6 days of onset [16]. It may also be that the subtypes of stroke were different among the enrolled patients. Our enrolled patients were diagnosed with AIS as confirmed by brain MRI or CT, while in the other studies, the enrolled patients had undergone percutaneous coronary intervention [17]. This study has some limitations that require consideration. First, the sample size of the enrolled patients in our study was small, and further studies are needed to validate our findings with a larger sample size. Second, information on the current medications of the enrolled patients was insufficient, because of which further research is needed to clarify other comorbidities and the medications of the enrolled patients. Third, we were unable to monitor the long-term prognosis of the patients. Further studies on this matter are recommended.

## 5. Conclusion

In summary, we provide the first evidence that the diagnosis and prognosis differ between patients with recurrent ischemic stroke and those with initial ischemic stroke. Plasma sLOX-1 levels are an independent prognostic marker in patients with recurrent AIS. Therefore, based on the history of stroke and strength of predictive factors, early and targeted intervention and secondary prevention should be conducted for patients with recurrent AIS. These measures are important in the prevention and treatment of recurrent ischemic stroke.

## Figures and Tables

**Table 1:** Baseline characteristics in patients with first-ever and recurrent stroke.

Baseline characteristics	All (*N* = 266)	Recurrent stroke (*N* = 101)	First-ever stroke (*N* = 165)	*p* value
Age (year)	63.0(56.0–73.5)	68.0(58.0–78.0)	61.0 (55.0–69.0)	0.013
Male, *n* (%)	196.0	76.0 (75.2%)	120.0 (72.7%)	0.65
Baseline systolic BP (mmHg)	150 (140–168.3)	150 (138.5–166.5)	150.0 (140.0–170.0)	0.5
Baseline diastolic BP (mmHg)	78.0 (89.0–94.3)	80.0 (77.0–93.0)	90.0 (80.0–95.0)	0.1
Time from onset (h)	3.0 (1.5–5.1)	2.8 (1.4–4.7)	3.0 (1.7–5.9)	0.55
Baseline NIHSS score	6.0 (3.0–11.0)	7.00 (4.0–13.0)	6.0 (3.0–10.0)	0.012
Clinical parameters (median)				
Neutrophil count (10^9^/L)	5.16 (3.95–6.85)	4.87 (3.90–7.49)	5.16 (3.98–6.54)	0.5
Lymphocyte count (10^9^/L)	1.53 (1.13–2.21)	1.37 (0.92–1.93)	1.71 (1.24–2.35)	0.006
Neutrophil-to-lymphocyte ratio	2.97 (2.13–5.55)	3.32 (2.18–6.62)	2.85 (2.11–4.67)	0.041
Platelet count (10^9^/L)	206.0 (170.5–243.5)	196.0 (161.0–224.5)	210.0(180.0–259.0)	0.055
Leukocyte count (10^9^/L)	7.66 (6.27–9.25)	7.66 (5.69–9.24)	7.66 (6.4–9.17)	0.966
Triglycerides (mmol/L)	1.51 (1.0–2.62)	1.39 (0.91–1.91)	1.69 (1.08–2.8)	0.025
Total cholesterol (mmol/L)	4.73 ± 1.19	4.35 ± 1.24	4.97 ± 1.12	0.000
High-density lipoprotein (mmol/L)	1.14 (0.97–1.40)	1.15 (0.97–1.39)	1.14 (0.97–1.40)	0.739
Low-density lipoprotein (mmol/L)	2.77 (2.20–3.46)	2.50 (1.77–3.30)	2.82 (2.38–3.60)	0.001
Risk factors, *n* (%)				
Hypertension	179	76 (75.2%)	103 (62.4%)	0.036
Diabetes mellitus	93	39 (38.6%)	54 (32.7%)	0.37
Hyperlipemia	72	32 (31.7%)	40 (24.2%)	0.21
Coronary heart disease	52	27 (26.7%)	25 (15.2%)	0.021
Atrial fibrillation	44	23(22.8%)	21 (12.7%)	0.14
Site of infarction (%)				0.72
Total anterior circulation (TAC)	17	5 (5%)	12 (7.3%)	
Partial anterior circulation (PAC)	199	76 (75.2%)	123 (74.5%)	
Posterior circulation (POC)	38	13 (12.9%)	25 (15.2%)	
Stroke etiologic subtypes (%)				0.89
Large artery atherosclerosis	151	57 (56.4%)	94 (57%)	
Small vessel disease	72	27 (26.7%)	45(27.3%)	
Cardioembolic	13	5 (5%)	8(4.8%)	
Other or unknown cause	1	0 (0%)	1(0.6%)	
Biomarkers (ng/mL), median				
sLOX-1 (pg/mL)	510.51 (314.53–877.60)	526.36 (339.32–826.61)	500.66 (288.44–915.42)	0.824

**Table 2:** Univariate logistic regression analyses for favorable outcome in patients with first-ever and recurrent stroke.

Univariable logistic regression				
	Recurrent stroke (*N* = 101)		First-ever stroke (*N* = 165)	
	OR (95% CI)	*p* value	OR (95% CI)	*p* value
Age (year)	1.064(1.024–1.105)	0.002	1.028(1.002–1.054)	0.035
Male, *n* (%)	1.24 (0.5–3.076)	0.642	1.705(0.844–3.444)	0.137
Baseline systolic BP (mmHg)	0.992(0.976–1.009)	0.387	0.999(0.986–1.012)	0.854
Baseline diastolic BP (mmHg)	1.001(0.972–1.032)	0.934	0.994(0.974–1.015)	0.598
Time from onset (h)	1.102(0.966–1.258)	0.15	1.056(0.981–1.136)	0.147
Baseline NIHSS score	1.201(1.105–1.306)	0.000	1.299(1.192–1.415)	0.000
Clinical parameters (median)				
Neutrophil count (10^9^/L)	1.087(0.954–1.239)	0.212	1.2 (1.058–1.362)	0.005
Lymphocyte count (10^9^/L)	0.726(0.438–1.202)	0.213	0.417(0.258–0.674)	0.000
Neutrophil-to-lymphocyte ratio	1.084(1.000–1.175)	0.05	1.3 (1.142–1.48)	0.000
Platelet count (10^9^/L)	0.994(0.986–1.003)	0.176	0.998 (0.993–1.003)	0.425
Leukocyte count (10^9^/L)	1.065(0.931–1.217)	0.36	1.104 (0.99–1.232)	0.076
Triglycerides (mmol/L)	0.966(0.807–1.156)	0.702	1.032(0.959-1.11)	0.404
Total cholesterol (mmol/L)	0.873(0.62-1.231)	0.439	0.968(0.729–1.286)	0.824
High-density lipoprotein (mmol/L)	1.042(0.278–3.901)	0.951	2.05(0.833–5.044)	0.118
Low-density lipoprotein (mmol/L)	0.983(0.633–1.528)	0.939	1.005 (0.722–1.4)	0.975
Risk factors, *n* (%)				
Hypertension	0.806(0.325–1.999)	0.642	1.312(0.669–2.576)	0.429
Diabetes mellitus	1.48(0.656–3.339)	0.345	1.874(0.954–3.68)	0.068
Hyperlipemia	0.556(0.233–1.327)	0.186	0.551 (0.246–1.23)	0.146
Coronary heart disease	1.544(0.636–3.749)	0.338	1.866 (0.789–4.412)	0.155
Atrial fibrillation	1.533(0.584–4.029)	0.386	2.773(1.131–6.802)	0.026
Site of infarction (%)	1.398(0.690–2.831)	0.353	0.749(0.418–1.343)	0.333
Total anterior circulation (TAC)				
Partial anterior circulation (PAC)				
Posterior circulation (POC)				
Stroke etiologic subtypes (%)	0.541 (0.2–1.468)	0.228	0.831 (0.416–1.66)	0.6
Large artery atherosclerosis				
Small vessel disease				
Cardioembolic				
Other or unknown cause				
Biomarkers (ng/mL), median				
sLOX-1 (per 100 pg/mL increase)	1.284 (1.123–1.470)	0.000	0.983 (0.919–1.05)	0.608

## Data Availability

The original data of this study are available from the corresponding authors upon reasonable request.
